# Comparative Analysis of the Chrysanthemum Leaf Transcript Profiling in Response to Salt Stress

**DOI:** 10.1371/journal.pone.0159721

**Published:** 2016-07-22

**Authors:** Yin-Huan Wu, Tong Wang, Ke Wang, Qian-Yu Liang, Zhen-Yu Bai, Qing-Lin Liu, Yuan-Zhi Pan, Bei-Bei Jiang, Lei Zhang

**Affiliations:** Department of Landscape Architecture, Sichuan Agricultural University, Chengdu, Sichuan, China; Institute of Genetics and Developmental Biology, Chinese Academy of Sciences, CHINA

## Abstract

Salt stress has some remarkable influence on chrysanthemum growth and productivity. To understand the molecular mechanisms associated with salt stress and identify genes of potential importance in cultivated chrysanthemum, we carried out transcriptome sequencing of chrysanthemum. Two cDNA libraries were generated from the control and salt-treated samples (Sample_0510_control and Sample_0510_treat) of leaves. By using the Illumina Solexa RNA sequencing technology, 94 million high quality sequencing reads and 161,522 unigenes were generated and then we annotated unigenes through comparing these sequences to diverse protein databases. A total of 126,646 differentially expressed transcripts (DETs) were identified in leaf. Plant hormones, amino acid metabolism, photosynthesis and secondary metabolism were all changed under salt stress after the complete list of GO term and KEGG enrichment analysis. The hormone biosynthesis changing and oxidative hurt decreasing appeared to be significantly related to salt tolerance of chrysanthemum. Important protein kinases and major transcription factor families involved in abiotic stress were differentially expressed, such as MAPKs, CDPKs, MYB, WRKY, AP2 and HD-zip. In general, these results can help us to confirm the molecular regulation mechanism and also provide us a comprehensive resource of chrysanthemum under salt stress.

## Introduction

Salinity is one of the most remarkable abiotic factors which limiting the productivity and growth of crop plants [1]. Salinity may cause a series of morphological, physiological and biochemical changes [2], such as decreasing the number of flower buds, reducing water absorption ability of root, enhancing programed cell death in some tissue types [1, 3]. Currently, increasing high salinity stress tolerance in plants is necessary. The genetic engineering strategies target at various metabolic pathways, accumulation of osmolytes, antioxidant enzymes and up-regulation of functional genes [4]. Among the complex molecular mechanisms, the up-regulation of functional genes expression occupies the important position in plant salt resistance [5, 6]. Various genes that function as stress sensors in signaling transduction pathways comprise a network of protein-protein reactions and transcription factors [7, 8]. Receptor-like kinase (RLK) [9], mitogen-activated protein kinase (MAPK) [10, 11], calcium-dependent and calmodulin-independent protein kinase (CDPK) [12] and sucrose non-fermenting-1-related protein kinase (SnRK) [13] have participated in abiotic or biotic stress signal pathways. What's more, according to DNA binding domain characteristics, transcription factors linked to stress response can be divided into several families, such as bHLH, AP2/EREBP, bZIP, NAC, WRKY, MYB and MYC [14, 15].

Chrysanthemum is a well-known ornamental plant in the world. It has large genomes and lacks the genomic information on the basis of salt tolerance [5]. Nowadays, the development of next-generation sequencing (NGS) technology has become a main approach for gene function annotation, gene expression analysis and identified numerous genes in fundamental molecular aspects related to stress response [16–18]. In particular, this method is not dependent on the existing genomic sequence [19]. Obviously, NGS has facilitated the molecular biology research in both model and non-model plant. The previous transcriptome sequencing analysis of plants under salt stress focused on *Arabidopsis thaliana* [20–22], rice [23, 24] and other model plants. Although transcriptional changes in model plants have been identified, there is very limited information about the molecular elements that are specific to non-model plants under salt stress. In recent years, transcriptome analysis of non-model species, such as *Eleusine coracana* [25], *Chrysanthemum nankingense* [26], *Populus euphratica* [27], has made progress in knowing about the molecular mechanisms under salt stress.

Due to the high frequency irrigation and high evapotranspiration, salinity conditions in the cultivation greenhouse seriously affected the quality and quantity of cut chrysanthemum [28]. We considered measure transcriptome composition of chrysanthemum and identified regulatory genes according to gene expression levels under salt stress. Besides, RNA-Seq technology has also provided an opportunity to understand the gene regulation networks. Ultimately, we obtained DETs between control and salt treated samples and identified salt-sensitive genes. Data obtained will not only be useful in identifying potential candidate genes influenced by salt stress, but also promoting genetic engineering of chrysanthemum to improve salt tolerance,

## Methods

### Plant materials and treatments

Chrysanthemum (*Chrysanthemum morifolium* (Ramat.) Tzvel.) 'jinba', a popular cut chrysanthemum cultivar which was used in this experiment. The seedlings were grown on MS medium with a thermoperiod of 25°C (day) and 22°C (night) and a photoperiod of 16 h. After 24 days, the seedlings subjected to salt treatment and concentration gradient of NaCl solution was used to slow down the salt injury: 100mM for the first day, 200mM for the second day and 400mM for three days. The control seedlings were watered daily. Finally, the leaves were sampled for subsequent RNA-Seq after 5 days treatment (leaf samples were frozen with liquid nitrogen and stored at -80°C). Three biological replicates were used in each treatment.

### RNA extraction, RNA-seq library construction and sequencing

Total RNA was extracted following the manufacturer's instructions. The quality and quantity of total RNA were assessed at an absorbance ratio (OD_260/280_ and OD_260/230_) and 1% agarose gel electrophoresis. Replicates were mixed to supply 2 pooled samples for the sequencing analysis. Strand-specific RNA-seq libraries were constructed according to Zhong etc. [29] and sequenced on Illumina HiSeq2000 system, Shanghai Sangon Biotech, Shanghai, China. The raw sequencing data have been submitted to the NCBI Sequence Read Archive (SRA) database with accession number SRP074167.

### Sequence analysis, *de novo* assembly and annotation

The raw reads were cleaned by removing adapter sequences, low quality sequences (reads with ambiguous bases 'N'), and reads with Q-value < 20 bases [25]. Then we discard the reads with length less than 35 bp. Next, *de novo* assembly was performed by using Trinity (release 20121005, http://trinityrnaseq.sourceforge.net/) software to generate unigenes [30]. In addition, the redundant duplicated reads were removed.

The resulting unigenes were blasted against NCBI Nr (NCBI non-redundant protein database), Swiss-Prot, TrEMBL, CDD (Conserved Domain Database), Pfam and KOG (euKaryotic Orthologous Groups) databases (E-value ≤ 1e-5) [31]. Functional annotation by GO (Gene Ontology database) was analyzed using the Blast2GO program [32]. The KEGG (Kyoto Encyclopedia of Genes and Genomes) was also used to predict and classify possible functions.

### Identification of differentially expressed genes

The RPKM reads (clean reads per kilobase per million) method (http://www.clcbio.com/manual/genomics/Definition_RPKM.html) was used to estimate transcript abundance on the base of eliminating the influence of different gene length and sequencing discrepancy [33, 34]. Furthermore, we used the method of an algorithm developed previous to compare the differences in gene expression [34, 35]. The false discovery rate (FDR) was applied to correct for P value in multiple hypothesis testing. And in this analysis, FDR ≤ 0.01 and an absolute value of log_2_ ratio ≥ 1 (two-fold change) were set as the threshold to judge the significance of gene expression differences [36]. The identified DETs were then carried out into GO functional analysis and KEGG pathway analysis.

### Quantitative Real-time PCR (qRT-PCR) validation of differential expression

In order to verify the data, eight salt responsive transcripts were selected to perform qRT-PCR with three replicates. The specific primers were designed using Primer 5 (Table 1). A 20 μL qPCR reaction mixture contained 10 uL SsoFast EvaGreen supermix (Bio-Rad, Hercules, CA, USA), 2 uL diluted cDNA sample, 300 nM of primers. And then performed qRT-PCR with the cycling conditions by using Bio-Rad CFX96^TM^ detection system: 95°C for 30 s, 40 cycles of 15 s at 95°C and 30 s at 60°C, in the end, the melting curve derived. Lastly, we used the 2^−ΔΔCT^ method to analyze the data and chose the *UBC* gene (Forward primer: CATCTACTCGTCAATCAGGGTT, Reverse primer: GTATGGGCTATCGGAAGGTC) as a reference [37].

**Table 1:** Primers of qRT-PCR for validation of the selected transcripts.

Gene ID	Description	Primers (5'-3')
comp94681_c0_seq1	MYB protein	F: GGTCATTGTCCAGATTCCACAR: CAGTGACCAGAAGCACCATAG
comp109545_c0_seq1	AP2 transcription factor	F: GAACCCATATACTAGACCCAAGTGR: GCCAAAATCAGCTCCCAAATC
comp69475_c0_seq1	transcription factor TGA	F: ATGGGTGAAGGTGGAAACTACR: CTTGTCGCATTGTCAAAATCCTAG
comp578479_c0_seq1	MADS-box transcription factor	F: AGAAAGGGTTTTGGGTGATACCR: CTCTTGGAAAATGTCACTTGTCTAC
comp119263_c0_seq4	WRKY transcription factor 33	F: CTCAAACACATCCTACAAATTCCCR: AGAAATGGGAAGTGAAGGTGG
comp114891_c0_seq1	ABA responsive element binding factor	F: ATTCTTGTCTGCCAGCTCAGR: TGAAGAGGGTGTATGTCTATTTCG
comp107847_c0_seq2	ATP-binding cassette. subfamily F. member 2	F: GATTTACCACAGCCATTCAACCR: CACTTACATTTCACGGGCATG
comp125136_c0_seq3	delta-1-pyrroline-5-carboxylate synthetase	F: CCCGTAGGAGTTGAAGGATTGR: CCCGAGAGCAGAGGATTTTAAG
comp126328_c0_seq23	6-phosphofructokinase 1	F: AAGTCTCAAGCACAGTACCACR: GAGCAGTATGGAGTTCGTGAG
comp110755_c0_seq1	unknown	F: CTTCAGAAATGGCATCACACGR: ATCGTGTCGGATGTCTTGTC

## Results and Discussion

### Sequencing and *de novo* assembly

**Fig 1:**
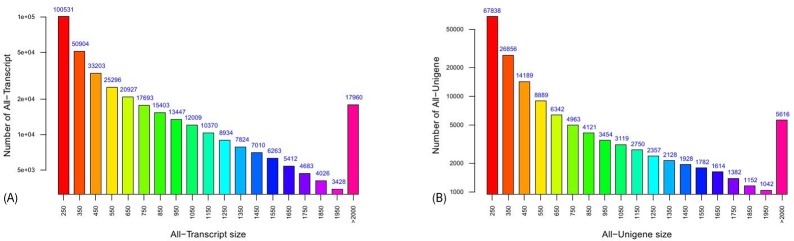
Overview of the chrysanthemum transcriptome sequencing. (A) Length distribution of chrysanthemum all-transcript. (B) Length distribution of chrysanthemum all-unigene.

NGS has been widely used for *de novo* transcriptome sequencing, especially in non-model organisms [38]. In this study, we established two cDNA libraries from chrysanthemum 'Jinba' under normal and salinity conditions, respectively. And a total of 10.0 Gb sequencing data were obtained by paired-end sequencing of Illumina Solexa RNA. Considering these reads have a substantially higher error rate, we filtered the raw data. The data retention rate was high and finally we got 94 million reads, the average length of which is 94.4 bp. After that, *de novo* assembly generated 365,323 unique transcripts (≥ 100 bp) with an average length of 732.0 bp and 161,522 unigenes (≥100 bp) with an average length of 575.7 bp from these reads. Length distribution of all-transcript and all-unigene are shown in Fig 1.

**Fig 2:**
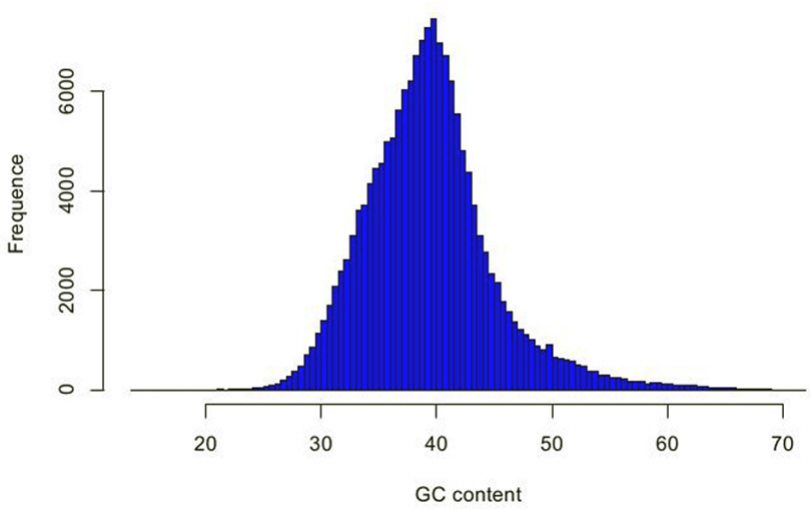
The frequency distribution of unigene GC content in chrysanthemum.

A total of 88,049 transcripts and 24,906 unigenes were greater than 1000 bp, and the N50 and N90 values were shown in Table 2. Among which, the longest transcript and unigene were all 12,403 bp. In addition, the GC content frequence distribution of unigenes is shown in Fig 2.

**Table 2:** The statistics of *de novo* assembly.

	All (> = 100bp)	> = 500 bp	> = 1000 bp	N50	N90	Total Length	Max Length	Min Length	Average Length
**Transcript**	365,323	180,947	88,049	1,080	312	267,422,176	12,403	201	732.02
**Unigene**	161,522	52,751	24,906	851	249	92,993,750	12,403	201	575.73

### Annotation of chrysanthemum transcript sequences

In contrast to different protein databases on the basis of amino acid sequence similarities, we had annotated the assembled transcripts of chrysanthemum. A total of 65,838 (40.8%), 40,075 (24.8%), 67,450 (41.8%), 34,614 (21.4%), 58,863 (36.4%) and 23,477 (14.5%) unigenes had significant hit in the NR, CDD, Swiss-Prot, Pfam, TrEMBL and KOG, respectively (Resemblance > 30% and E-value ≤ 1e-5). The similarities of genes were mainly based on BLASTx.

**Fig 3:**
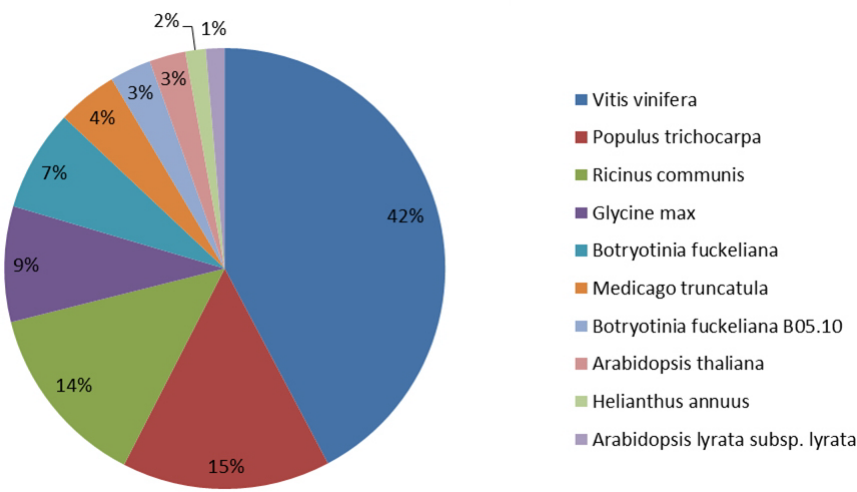
The percentage of the chrysanthemum unigenes that showing similarity to genes in other plant species. The chrysanthemum unigenes aligned by BLASTx to the Nr database.

To gain insight into the percentage of the chrysanthemum unigenes that showing similarity to genes in other plant species, we performed species distribution of chrysanthemum in NR database, in which 76.5% genes were annotated with the largest number of 10 species (Fig 3). Among the various plants, the chrysanthemum unigenes had the maximum number of homologous to *Vitis vinifera* (42.0%) while the *Populus trichocarpa* and *Ricinus communis* were 15.0% and 14.0%, respectively. Interestingly, these high similarities have also been described in the study of *chrysanthemum nankingense* (Asteraceae) [26]. And *Glycine max* (9.0%), *Botryotinia fuckeliana* (7.0%), others (13.0%) were in the following. These results may demonstrate that chrysanthemum has closer relationship with *Vitis vinifera* while *Arabidopsis lyrata subsp*. *lyrata* is further.

**Fig 4:**
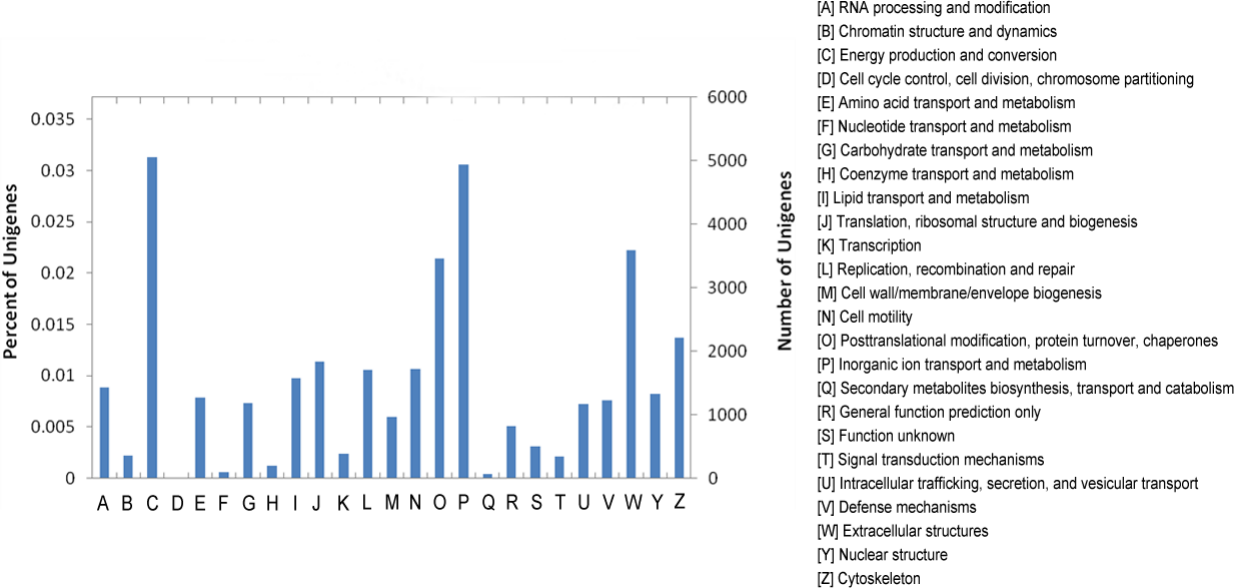
Cluster of orthologous groups for enkaryotic complete genomes (KOG) classification. A total of 23,477 sequences with KOG classifications within the 25 categories are shown.

We further classified the functions by using the KOG database. 23,477 unigenes were assigned with 25 specific KOG categories, which were listed in Fig 4. The top three categories were "signal transduction mechanisms" with 5,061 unigenes (21.6%), "posttranslational modification, protein turnover, chaperones" with 4,938 unigenes (21.0%) and "general function prediction" only with 3,587 unigenes (15.3%). The KOG categories related to stress response are as follows: "defense mechanisms" (0.8%), "secondary metabolites biosynthesis, transport and catabolism" (7.8%), "inorganic ion transport and metabolism" (3.5%), and "lipid", "coenzyme", "carbohydrate", "nucleotide", "amino acid" transport and metabolism were 18.9% in total.

**Fig 5:**
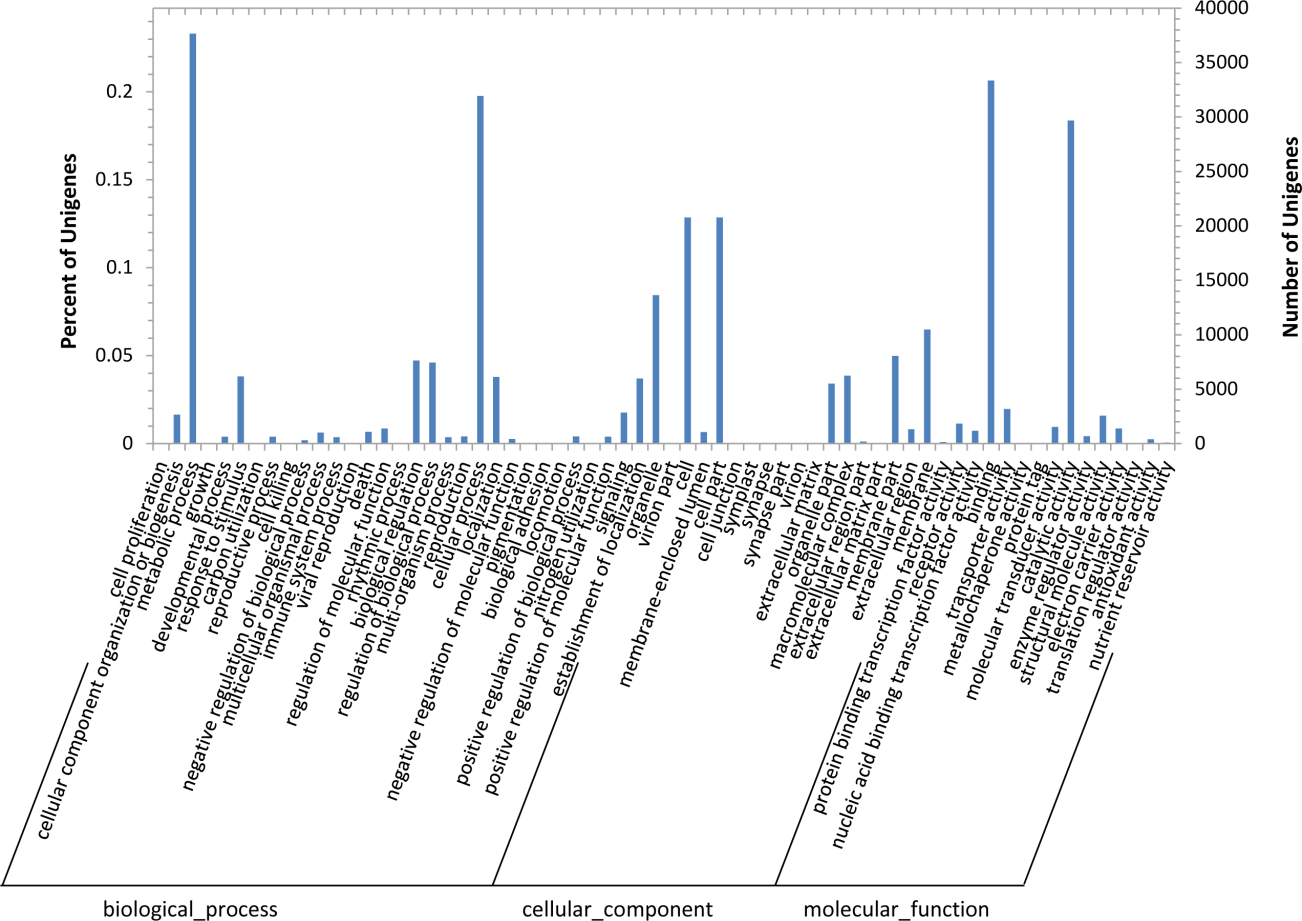
Chrysanthemum functional classification by gene ontology (GO) analysis. A total of 12,153 unigenes have been assigned GO terms and they are classified into three GO categories: biological process, cellular component, and molecular function.

The GO analysis allowed the functional classification of 12,153 unigenes. As shown in Fig 5, among which 41.7% were assigned in the "biological process category", 31.3% in the "cellular component category", and 27.0% in the "molecular function category". The major classes of biological process among the comparison of GO classification were "metabolic process", "cellular process", "response to stimulus", "biological regulation" and "regulation of biological process". The predominant cellular components were "organelle", "cell", "cell part" and "membrane". And for molecular function were "binding", "catalytic activity", "electron carrier activity", "structural molecule activity" and "transporter activity".

Additionally, we predicted biochemical pathways from the KEGG database, in which, a total of 25,173 (15.6%) unigenes were grouped into 306 pathways. KEGG pathway analysis showed that the genes were mainly located in "ribosome" (ko03010, 2001 unigenes), "protein processing in endoplasmic reticulum" (ko04141, 727 unigenes), "RNA transport" (ko03013, 618 unigenes) and "spliceosome" (ko03040, 616 unigenes). Other important pathways related to abiotic stress include "arginine and proline metabolism" (ko00330), "plant hormone signal transduction" (ko04075), "glutathione metabolism" (ko00480), "flavonoid biosynthesis" (ko00941), "carotenoid biosynthesis" (ko00906) and so on.

### Identification of DETs under salt stress in chrysanthemum

**Fig 6:**
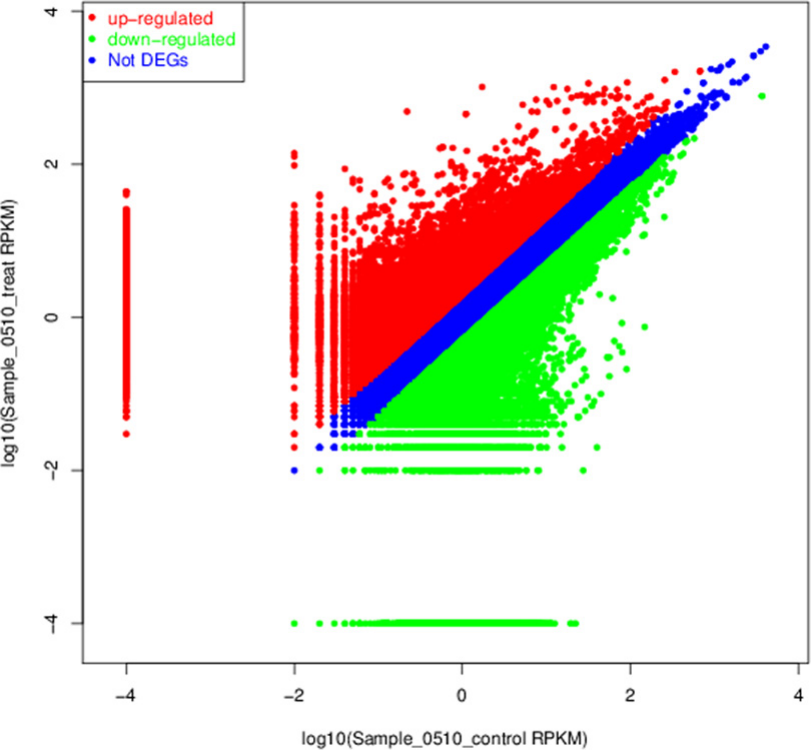
The gene expression level of chrysanthemum between the two libraries (Sample_0510_control and Sample_0510_treat). Red dots represent transcripts more prevalent in the library of salt-treated one, green dots show those present at a lower frequency in salt-treated chrysanthemum and blue dots indicate transcripts that did not change significantly.

Here, we used the method of sequence similarity alignment to calculate the expression abundance of each transcript in two samples. Moreover, the differential expression analysis was made according to the expression abundance value. In total, we identified 126,646 DETs between salt-treated and control samples, and there were 60,685 DETs up-regulated and 65,961 down-regulated under salt stress. Based on the value of expression abundance (RPKM), the gene expression levels of two samples are shown in Fig 6.

**Fig 7:**
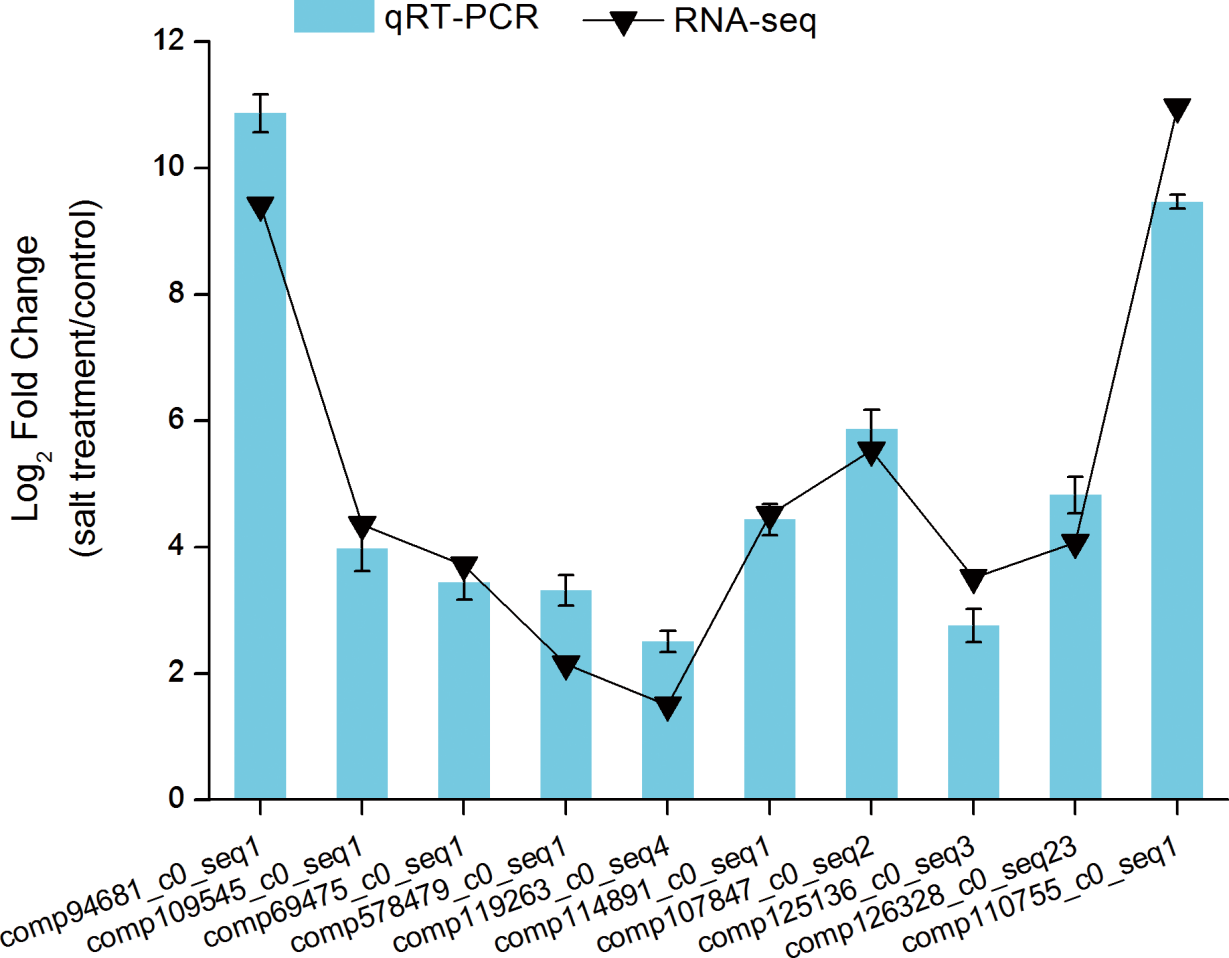
The relative expression levels of ten DETs between RNA- seq and qRT-PCR in chrysanthemum. The relative gene expression levels were determined by 2^−ΔΔCT^ as expressed. Transcript levels were normalized to the expression level of *UBC*. *Error bars* SE from three independent replicates.

To further evaluate the reliability of the Illumina sequencing results, ten differential expression transcripts were selected to perform qRT-PCR based on their predicted functions, such as transcription factors (MADS, MYB, WRKY, AP2, TAG), a salt transporter (ATP-binding cassette (ABC) transporters), a hormone responsive factor (ABA responsive element binding factor), a key regulatory enzyme involved in the biosynthesis of proline (*P5CS*, delta-1-pyrroline-5-carboxylate synthetase), a gene encoding *PFK1* (6-phosphofructokinase 1). As can be seen, there was one gene with unknown function. The result of qPCR experiment and RNA-seq were consistent (Fig 7).

### The enrichment analyses of DETs by GO and KEGG databases

The enrichment analysis of chrysanthemum transcripts by GO database help us to realize the function of these DETs and 638 GO terms were enriched (P value ≤ 0.01). "ATP binding" (9,005 DETs), followed by "membrane" (7,086 DETs), "integral to membrane" (6,113 DETs), "oxidation-reduction process" (5,244 DETs) and "metabolic process" (5,041 DETs). Other highly enriched GO terms related to stress were "oxidoreductase activity", "defense response", "protein kinase activity" and "protein serine/threonine kinase activity". This consequence indicated that the GO terms including DETs might occupy an important position in the molecular mechanism of salt resistance in chrysanthemum. Plant hormones have a functional role in plant response to biotic and abiotic stresses [39]. In our study, GO terms related to plant hormones including "abscisic acid biosynthetic process", "gibberellin 3-beta-dioxygenase activity", "jasmonate O-methyltransferase activity", "ethylene biosynthetic process", "cytokinin metabolic process" and "brassinosteroid biosynthetic process", all of which were enriched both in up-regulation and down-regulation. The former finding implied that plant secondary metabolites are widely involved in plant physiological processes, such as growth, development and defense [40, 41]. Nevertheless, GO terms related to plant secondary metabolites in this study including "terpenoid metabolic process", "flavonoid biosynthetic process", "lignin catabolic process", "organic acid metabolic process" and "alkaloid metabolic process" and so on. Furthermore, GO term "chlorophyll catabolic process" was up-regulated while "chlorophyll biosynthetic process" was down-regulated. This may be due to the damage of plant photosynthesis mechanism under salt stress.

In addition, we confirmed pathways by mapping DETs to terms in the KEGG database. 92 pathways were significantly enriched (P value ≤0.01) among the all 278 differentially expressed pathways. "Ribosome" was the most enriched pathway (1,821 DETs). Other crucial pathways related to abiotic stress including "plant hormone signal transduction", "sesquiterpenoid biosynthesis", "flavonoid biosynthesis", "alpha-Linolenic acid metabolism", "phenylpropanoid biosynthesis" and other pathways.

### Potential biological pathways influenced by salt stress

We analyzed biochemical pathways influenced by salt stress on the base of our expression profiling. The major identified pathways in chrysanthemum are shown in S1 Table.

The accumulation of flavonoids is a hallmark of plant stress [42]. In this study, there were 133 DETs with KEGG annotation in flavonoid biosynthesis pathway. In addition, signal-transduction cascades mediate the sensing and processing of stimuli [43], and "MAPK signaling pathway", "Calcium signaling pathway", "PPAR signaling pathway" and "Plant hormone signal transduction" were all annotated DETs.

In plants, carotenoids are mainly found in the chloroplasts of plants and chromoplasts of the flowers and fruits, the two important functions in plant photosynthesis of which are participating in light absorption and preventing photooxidation in precursor cells [44, 45]. Meanwhile, carotenoids are also signaling molecule precursors of plants and can respond to outside stimulus [46]. Some reports showed that carotenoids not only can participate in the process of plant hormones biosynthesis, such as abscisic acid and strigolactone [47], but also the synthesis of defense chemicals [48]. Carotenoids can consider as a scavenger of free radicals and then reduce the hurt from stress [48]. In our study, the key enzyme "phytoene synthase" (crtB, EC:2.5.1.32), "beta-ring hydroxylase" (crtR-b, EC:1.14.-.-) and "beta-carotene 3-hydroxylase" (crtZ, EC:1.14.13.129) in the "carotenoid biosynthesis pathway" were found up-regulated. In rapeseed and tomato, phytoene synthase has been demonstrated to be the rate-limiting enzyme in carotenoid biosynthetic [49, 50]. The increasing of phytoene synthase content provided a sufficient amount of synthetic substrate, so that the β-carotene and zeaxanthin accumulated, and then presented up-regulated. In carotenoid biosynthesis pathway, 9-*cis*-epoxycarotenoid dioxygenase is the direct substrate for phytohormone abscisic acid (ABA) synthesis [51]. We found that "abscisic acid 8'-hydroxylase" (EC:1.14.13.93), "abscisic-aldehyde oxidase" (EC:1.2.3.14) and "9-*cis*-epoxycarotenoid dioxygenase" (EC:1.13.11.51) were all up-regulated. Thus, changes of carotenoid content and composition can lead to changes in plant physiology and biochemistry.

### Sensing and signaling genes involved in the response to salt stress

Ionic signaling pathway is an important component of salt stress signal transduction, and plant salt tolerance is related to the expression and activity changes of ion transporters to some extent, such as Na^+^, K^+^, H^+^ and Ca^2+^ [6]. Besides, the change of V-ATPase activity determines the survival of plant cells under stress [52]. Among the ion transport genes, 16 Ca^2+^/H^+^ antiporter (12 up-regulated and 4 down-regulated), 2 Na^+^ symporter (all up-regualted) and 111 V-type H^+^-ATPase (75 up-regulated and 36 down-regulated) were differentially expressed,

Ca^2+^ is one of the very important ubiquitous second messengers in signal transduction pathways and usually its concentration increases in response to the stimuli including stress signals [53]. In previous studies, genes related to Ca^2+^ signaling pathway also maintain an important role in different environmental stresses response of rice [54], *Arabidopsis thaliana* [55], *Chrysanthemum nankingense* [36] and so on. In total, there were 51 genes found to be up-regulated while 100 found to be down-regulated in Ca^2+^ signaling pathway (S2 Table). Ca^2+^ signaling pathway include many members, such as calmodulin, calcium exchanger, calcium-binding protein, calcium dependent protein kinases (CDPKs) and CDPKs are unique Ca^2+^ sensors in higher plants [54]. What's more, *AtCPK4* and *AtCPK11* [56], *OsCDPK7* [57] were reported to confer stress tolerance. There were 28 CDPKs up-regulated and 18 down-regulated (log_2_ fold change ≥ 1) in this study. Salt stress sensing and signaling genes also influenced by other protein kinases [58]. The major differentially expressed protein kinases are shown in S3 Table. The mitogen activated protein kinases (MAPKs) can be activated under abiotic stresses in plants [59]. The MAPK cascade itself is shaped by three major constituents, MAPK kinase kinase (MAP3K), MAPK kinase (MAP2K) and MAPK [60]. The MAPKs, such as *ZmSIMK1* [61], *OsMPK44* [62], MKKK20 [63], are related to plant salt tolerance. There were 12 up-regulated (log_2_ fold change ≥ 1) and 34 down-regulated genes (log_2_ fold change ≤ -1) involved in the MAPK cascade. Among which, only 6 *MAPKKK* and 3 *MAPKK* genes were up-regulated, and all three *MAPK* genes were up-regulated. Protein phosphatase 2C (PP2C) which is a Mn^2+^- or Mg^2+^- dependent protein serine/ threonine phosphatase act as a key player in plant ABA signal transduction processes [64, 65]. In this study, we identified 121 differentially expressed serine/threonine-protein kinase genes. And there were 80 *PP2C* genes up-regulated (log_2_ fold change ≥ 1) and only 7 down-regulated (log_2_ fold change ≤ -1). Above all, there were 6 *PP2C* genes significantly differentially expressed (log_2_ fold change ≥ 10). The differentially expressed PP2Cs are shown in S4 Table.

#### Salinity-responsive transcription factors

Transcription factors act as upstream regulators to regulate gene expression in metabolic pathways [15]. In this study, we identified more than six major transcription factor (MYB, MYC, WRKY, AP2/ERF, HD-zip, E2F, zinc finger and so on) that were known to be stress-related genes. The major up or down regulated transcription factors are shown in S5 Table. MYB proteins were the largest group in our study, which play regulatory roles in developmental processes and defense responses in plants [66] and have been found in *Arabidopsis* [66], wheat [67], rice [66] and other plants. In our DETs data, there were 126 up-regulated and 92 down-regulated MYB transcripts. And other researches have also reported that WRKY [68], AP2/ERF [69] and HD-zip [70] transcription factors together mediated salt response. *TOC1* is a member of pseudo response regulator (PRR) family and *TOC1* is a multi-domain signaling component of the plant circadian clock [71]. *TOC1* regulates the diurnal expression of the ABA-related gene *ABAR*/*CHLH*/*GUN5* by direct binding to its promoter [72]. 13 up-regulated PRR family genes were found including a *TOC1* homologue in our study. TGA proteins were found to be up (18) or down (16) regulated in this paper and that TGA protein is a member of bZIP transcription factor which regulates the expression of some stress- responsive genes [73]. These differentially expressed genes may provide help for the study of salt tolerance in chrysanthemum.

## Conclusions

The current study focuses on identifying the major differentially expressed genes in chrysanthemum during salt stress response using Illumina sequencing technology and validating them by qRT-PCR analysis. DEGs were identified in chrysanthemum under salt stress, including sensing and signaling genes, protein kinases and transcription factors. Functional annotation and classification, metabolic pathway analysis provided their function in possible regulation of salinity adaptation in chrysanthemum. These results provide valuable data for the molecular mechanisms to study the underlying salt tolerance and may be the basis of further research on gene regulatory networks of chrysanthemum.

## Supporting Information

S1 Table The major identified pathways in chrysanthemum. (XLSX)

S2 Table The differentially expressed genes involved in Ca^2+^ signaling pathway. (XLSX)

S3 Table The major differentially expressed protein kinases in chrysanthemum. (XLSX)

S4 Table The differentially expressed PP2Cs in chrysanthemum. (XLSX)

S5 Table The major differentially expressed transcription factors in chrysanthemum. (XLSX)
